# The role of vascular endothelium and exosomes in human protozoan parasitic diseases

**DOI:** 10.20517/2574-1209.2020.27

**Published:** 2020-09-27

**Authors:** Sanjay Varikuti, Bijay Kumar Jha, Erin A. Holcomb, Jodi C. McDaniel, Manjula Karpurapu, Nidhi Srivastava, Bradford S. McGwire, Abhay R. Satoskar, Narasimham L. Parinandi

**Affiliations:** 1 Department of Pathology, The Ohio State University Medical Center, Columbus, OH 43201, USA; 2 Department of Bioscience & Biotechnology, Banasthali University, Banasthali 304022, India; 3 Division of Infectious Diseases, Department of Internal Medicine, The Ohio State University Medical Center, Columbus, OH 43201, USA; 4 College of Nursing, The Ohio State University, Columbus, OH 43201, USA; 5 Division of Pulmonary, Critical Care, and Sleep Medicine, Department of Internal Medicine, The Ohio State University Medical Center, Columbus, OH 43201, USA

**Keywords:** Vascular endothelium, exosomes, leishmaniasis, malaria, trypanosomiasis, toxoplasmosis

## Abstract

The vascular endothelium is a vital component in maintaining the structure and function of blood vessels. The endothelial cells (ECs) mediate vital regulatory functions such as the proliferation of cells, permeability of various tissue membranes, and exchange of gases, thrombolysis, blood flow, and homeostasis. The vascular endothelium also regulates inflammation and immune cell trafficking, and ECs serve as a replicative niche for many bacterial, viral, and protozoan infectious diseases. Endothelial dysfunction can lead to vasodilation and pro-inflammation, which are the hallmarks of many severe diseases. Exosomes are nanoscale membrane-bound vesicles that emerge from cells and serve as important extracellular components, which facilitate communication between cells and maintain homeostasis during normal and pathophysiological states. Exosomes are also involved in gene transfer, inflammation and antigen presentation, and mediation of the immune response during pathogenic states. Protozoa are a diverse group of unicellular organisms that cause many infectious diseases in humans. In this regard, it is becoming increasingly evident that many protozoan parasites (such as *Plasmodium*, *Trypanosoma*, *Leishmania*, and *Toxoplasma*) utilize exosomes for the transfer of their virulence factors and effector molecules into the host cells, which manipulate the host gene expression, immune responses, and other biological activities to establish and modulate infection. In this review, we discuss the role of the vascular endothelium and exosomes in and their contribution to pathogenesis in malaria, African sleeping sickness, Chagas disease, and leishmaniasis and toxoplasmosis with an emphasis on their actions on the innate and adaptive immune mechanisms of resistance.

## INTRODUCTION

### The vascular endothelium

The vascular endothelium (VE) is a large endocrine organ consisting of a single layer (one cell thick) of endothelial cells (ECs). The VE mediates regulatory functions in cell proliferation, angiogenesis, permeability, blood flow, thrombosis, thrombolysis, coagulation, homeostasis, and inflammation^[1,2]^. It also acts as the barrier between vascular and parenchymal compartments of all organs and regulates the exchange of gases, immune cell trafficking, metabolism, and the spread of infections. The entire circulatory system, from the heart to the smallest capillaries, is layered with ECs, which carry out unique functions such as fluid filtration, maintaining the tone of the blood vessel, platelet and leukocyte interactions, neutrophil recruitment, and hormone trafficking^[3,4]^. The VE plays a major role in leukocyte recruitment from the vessel lumen and transit into tissue parenchyma^[5,6]^, and it is also the precursor for both hematopoietic and endothelial lineages^[7]^. The role of the VE is governed by the presence of many membrane-bound receptors for molecules such as proteins, lipid transporting particles hormones, and metabolites^[8,9]^. Recent studies have identified the immunological role of the VE from the secretion of cytokines and chemokines to the expression of adhesion molecules and antigen presentation^[10–12]^.

The VE is also involved in bacterial, viral, and protozoan infectious disease processes. However, the interactions between the pathogen and the VE and how they both influence the disease outcome needs to be explored further. A clearer understanding of these relationships may help in identifying potential targets for therapeutic intervention(s) to prevent and/or reduce disease severity.

### Role of the VE in innate immunity

The VE serves as the first line of defense against the physical stimuli and chemical agonists that are present in the bloodstream during infection and disease by activating the inflammatory response when receptors on the ECs, such as the toll-like receptors (TLRs) and NOD-like receptors (NLRs), recognize Pathogen associated molecular patterns (PAMPs), damage associated molecular patterns (DAMPs), and pro-inflammatory cytokines such as interleukin (IL)-1β or TNF-α^[13,14]^. These recognition signals regulate the expression of pro-inflammatory genes (IL-1, IL-6, TNF-α, and IFN-γ), leukocyte recruitment, phagocytosis, and a subsequent adaptive immune response^[14]^. During the early phase of an inflammatory response, ECs are stimulated to release nitric oxide (NO), prostacyclin-2 (prostaglandin I2 or PGI_2_), and endothelin-1 to increase vasodilation by relaxing the surrounding smooth muscle cells^[13,15]^. Capillary permeability is further facilitated by the removal of occludin between EC junctions and inflammatory mediators such as kinins, cytokines, histamine, arachidonic acid, and complement components produced during this onset of inflammation^[14]^. VE activation upregulates expression of adhesion molecules such as intercellular adhesion molecule-1 (ICAM-1), vascular cell adhesion molecule-1 (VCAM-1), and E-selectin by ECs to aid chemokine-guided leukocyte rolling and extravasation through the vessel wall into the target site of the tissue .^[14]^

### Role of the VE in adaptive immunity

The adaptive immune response can be mediated by the VE in multiple ways under certain conditions. For example, thrombin-activated platelets as discussed above, upregulate CD40L similar to activated T cells and are therefore able to interact with CD40 on the EC surface to promote cytokine and chemokine secretion, adhesion molecule expression, tissue factor release, and leukocyte recruitment^[16,17]^. Furthermore, the upregulation of adhesion molecules and chemokines by activated ECs also serves to selectively recruit specific T cell types in circulation^[18]^. During this recruitment, antigen presentation may occur between the ECs and T cells mediated by major histocompatibility complex (MHC). It is known that ECs express MHC Class I on their surface, as well as MHC Class II in certain vasculatures of the body^[14]^. Due to this presence of MHC, ECs can act as antigen-presenting cells (APCs) to present antigen to effector or memory T cells^[19]^. Interaction with T cell co-stimulatory or co-inhibitory molecules expressed on ECs, such as CD80, CD86, LFA-3, ICOS-L, PDL-1, CD40, and CD134L, also mediates the T cell response^[14]^.

Due to their ability to act as APCs, ECs may generate certain T cell subsets to induce either inflammation or immune tolerance^[14]^. For instance, it has been shown that ECs support T cell proliferation and increases in the number of suppressor Treg cells^[20]^ and can stimulate the production of pro-inflammatory Th17 cells under inflammatory conditions^[21]^. ECs are also directly recognized by effector memory CD4+ T cells to stimulate IFN-γ production and subsequent CD4+ Th1 polarization^[22]^. This phenomenon has been further supported by a study involving activation of the ECs mediated by the C3a and C5a anaphylatoxins, which induce a Th1 phenotype, an increase in IFN-γ production, and activation of B lymphoblasts^[23]^. Taken together, the evidence suggests that VE plays a critical role in the initial onset of inflammation, innate immunity, and subsequent adaptive immunity in response to diverse stimuli present within the bloodstream.

### Exosomes

Exosomes are membrane-bound extracellular vesicles between 40 and 100 nm in diameter that have been recognized as important players in cell-to-cell signaling^[24]^. They are secreted into the extracellular space by ECs and various other cells, such as platelets, immunocytes, and smooth muscle cells^[25–27]^ and are present in almost all biological fluids^[28]^. Extracellular exchange of exosomes containing various bioactive molecules takes place continuously between organelles to foster communication during homeostasis and diseased states^[29]^. Exosome production begins with the internalization of the cellular membrane through endocytosis to form an endosome, followed by the invagination of the endosomal membrane, which matures into multivesicular bodies (MVBs). The MVBs can then either be degraded by internal lysosomes or are transported to the cell membrane to undergo transcytosis or fusion and release of the contained liberates intraluminal vesicles into the extracellular space, becoming exosomes^[28,30]^. Contents carried by exosomes consist of different proteins, lipids, metabolites, RNA, and DNA, which may be exchanged between the exosomes and their target cells.

Originally, exosomes were thought to be involved only in the process of excretion of unnecessary/unwanted proteins, and several studies have revealed that cellular stress triggers an enhanced release of exosomes^[31–34]^. However, further studies have suggested the participation of exosomes in cellular communication associated with many physiological and pathological states due to their ability to influence the phenotype of recipient cells^[24,28]^. Exosomes can target cells through specific receptor binding to activate cell-to-cell signaling pathways, which induce specific functions^[35]^. Information from an exosome may be transferred to the recipient cell either by interacting at the cell surface or by endosomal uptake^[36]^. Functions of released exosomes include horizontal gene transfer, inflammation, antigen presentation, tumor progression, and mediation of the immune response during pathogenic states ^[35,37]^.

Exosomes have been isolated from protozoa, bacteria, viruses, and fungi but each has distinct exosome profiles with varying compositions^[38]^. Protozoan parasites modulate host cells by producing exosomes containing virulence factors and effector molecules. In this way, parasites can manipulate host gene expression, immune responses, and other factors that favor parasite growth, survival, and pathogenesis^[39]^. Exosomes released during infection may also facilitate host immunity^[38,40]^.

### Role of exosomes in innate immunity

Exosomes are thought to play an important role in the host immune response to infection. Reciprocal cross-talk between platelets and neutrophils is enabled by neutrophil-derived exosome transfer of arachidonic acid to platelets, which is enzymatically converted into thromboxane A2 (TxA2). Release of TxA2 from platelets contributes to the upregulation of ICAM-1 on neutrophils, which binds to the EC surface and enables their extravasation to the site of infection to engage microbes^[41]^.

Exosomes are directly involved in immune signaling in as much as they can cargo pro-inflammatory and anti-inflammatory cytokines to target cells^[42–45]^ as well as stimulate the secretion of these cytokines from recipient cells^[35,46,47]^. For instance, a recent study documented the role of apoptotic exosome-like vesicles in promoting the synthesis of the pro-inflammatory IL-1β in macrophages, thereby contributing to their activation^[48]^. In contrast, exosomes have also been shown to promote the production of the anti-inflammatory transforming growth factor-β (TGF-β) in macrophages leading to the inhibition of the innate immune response^[47,49]^. Murine LPS-stimulated macrophages produce exosomes containing endoplasmic reticulum aminopeptidase 1, TNF-α, IFN-γ, and CCL3, which induces phagocytosis and nitric oxide synthesis in adjacent recipient macrophages^[47,50]^, important cellular mediators in clearing the microbial infection. Additionally, epithelial cell uptake of exosomes secreted by LPS-induced dendritic cells (DCs) has been shown to stimulate their activation and subsequent release of cytokines and chemokines to further the innate response^[51,52]^.

PAMPs are vital for recognition of pathogens and activation of immune cells and have been found inside exosomes secreted by both infected cells^[53,54]^ and pathogens^[55–57]^. Interaction of PAMP-containing exosomes with the innate immune cells can induce inflammation. This has been observed in bacteria-infected macrophages, which release exosomes-contained pathogen antigens, which in turn promote maturation of DCs and secretion of pro-inflammatory cytokines^[58]^.

### Role of exosomes in adaptive immunity

Exosomes not only influence innate immune responses but can also play a marked role in adaptive immunity by promoting immune activation or suppression. B-lymphocytes infected with Epstein-Barr virus were the first immune cells discovered to release exosomes. These exosomes were shown to contain peptide-bound MHC Class I and II, B7 co-stimulatory, and ICAM-1 adhesion molecules that could induce a specific T cell response through the direct presentation of MHC Class II antigen^[51]^. Later, DCs were also found to release exosomes, possessing MHC Class I and II and T cell co-stimulatory molecules, leading to direct antigen presentation and CD4+ and CD8+ T cell activation. DC-derived exosomes can also mediate antigen presentation through bystander DCs, by either cross-dressing or internalization of the exosomes and subsequent peptide transfer to the recipient cell MHC molecules. It is known that immature DCs have decreased T cell activation ability due to the lack of co-stimulatory molecules but do produce more antigen-presenting exosomes than mature DCs. Therefore, the transfer of exosomes from antigen-containing donor DCs to recipient bystander DCs allows indirect antigen presentation and activation of T cells, especially from the immature DCs, which are unable to successfully present the antigen on their own^[51,59]^.

Furthermore, the CD4+ and CD8+ T cells can also constitutively secrete exosomes containing TCR/CD3 complexes, which is enhanced upon TCR activation^[60]^ and can be either immune-activating or immune-suppressing depending on the microenvironment. Activated T cells can transfer exosomes to induce activation of resting T cells and enhance adaptive immune responses. For instance, CD3+ T cells activated with IL-2 and anti-CD3-secreted exosomes promote the proliferation of CD8+ T cells as well as their subsequent cytokine secretion^[61]^.

Exosomes can also promote an immunosuppressed environment under certain circumstances. Exosomes produced by immature DCs have been shown to induce T cell anergy/deletion as well as activate CD4+ regulatory T (Treg) cells^[38]^. Treg cells promote an immunosuppressive environment by secreting exosomes containing anti-inflammatory molecules that inhibit IFN-γ secretion and CD4+ Th1 cell proliferation, as well as signal other T cells to differentiate into Treg cells. It has also been found that CD4+ T cells and certain B cells whose exosomes contain FasL can induce apoptosis in recipient T cells. Furthermore, ECs transfer anti-inflammatory miRNA through exosomes to mediate T cell responses and prevent chronic inflammation^[62]^.

## HUMAN PROTOZOAN PARASITIC DISEASES

In humans, protozoan parasitic infections represent a substantial threat causing more than one million deaths annually^[63]^. According to the World Health Organization (WHO), it is estimated that protozoan parasitic infections occur in billions of people worldwide and are associated with significant mortality and morbidity and negatively impact many countries economically (WHO.org and^[64]^). The three most important protozoan diseases in humans are malaria, leishmaniasis, and African trypanosomiasis that cause disability-adjusted life years in millions of patients (WHO.org 2008 and 2010).

Many protozoan infections cause non-self-limiting chronic infections and neglected diseases. There are 20 diseases that affect more than one billion people in almost 149 tropical and subtropical countries and are responsible for approximately 12% of the total global health burden, which are categorized as Neglected Tropical Diseases (NTDs)^[65]^ (https://www.who.int/neglected_diseases/diseases/en/). Due to the disease burdens, limited available effective treatments, and lack of vaccines, the WHO has classified NTDs such as leishmaniasis, Chagas disease, and Human African trypanosomiasis under the specialized program “Innovative and Intensified Disease Management”^[66]^. While most of these infections occur in developing countries, it is evident that developed countries are also affected^[67]^. In addition, the emergence of anti-microbial resistant strains, toxicity, and low effectiveness of the currently available treatments pose a substantial problem^[68]^. Globalization and socioeconomic conditions also play a major role in the emergence and spreading of specific protozoan parasitic infections^[69]^. Furthermore, while many protozoan infections remain asymptomatic, they can lead to death, especially among children. It is important to note that, unlike bacterial and viral infections, many protozoan parasitic infections do not have readily available vaccines^[70]^. The lack of reliable drugs, difficulties in vector control, and limited knowledge about these infections are also major barriers to preventing, controlling, and treating these protozoan parasitic infections. Understanding the host immune response to protozoan parasites is very important to develop vaccines and new drugs with low toxicity and high efficacy.

The most severe protozoan infections, such as malaria, leishmaniasis, Chagas diseases, Human African trypanosomiasis (HAT), and toxoplasmosis are transmitted through blood, although Chagas disease and Toxoplasmosis can also be transmitted through the consumption of infected meat and liquid^[71]^. Hematophagous arthropod vectors serve as intermediate hosts and transmit the parasites (*Leishmania*, *Trypanosomes*, and *Plasmodium*) between successive vertebrate hosts. Thus, these bloodborne and vector-transmitted infections involve complex interactions between the parasite and insect and mammalian hosts.

Below, we discuss the role of VE and exosomes in malaria, leishmaniasis, toxoplasmosis, Chagas disease, and HAT, as well as their effects on the innate and adaptive immune responses in these infections. The roles of VE and exosomes in these infections are summarized in Tables 1 and 2, respectively.

## MALARIA

Malaria is the most prevalent tropical disease, which annually infects 300-500 million individuals worldwide (CDC.org). It is estimated that 2-3 million people are at risk every year, most of whom are children who die from the infection without the proper treatment along with the development of other complications. Malaria is prevalent among 90 countries, which represent 40% of the worlds population^[125]^. Malaria is caused by an intracellular protozoan parasite of the genus *Plasmodium*, in which five species are known to infect humans: *P. falciparum*, *P. malariae*, *P. ovale*, *P. vivax*, and *P. knowlesi* ^[126,127]^. *P. falciparum* causes the most severe clinical form of the infection leading to major morbidity and mortality. Female Anopheles mosquitoes transmit this disease to humans when releasing sporozoites while taking a blood meal. These circulate in the blood and invade and mature in hepatocytes. Hepatic forms are released and invade erythrocytes where merozoites further increase in number and are released and invade other red blood cells (CDC.org: https://www.cdc.gov/malaria/about/disease.html).

### Role of the VE in malaria

The VE plays a major role in host-parasite interactions and the severity of the malarial disease. It has been shown that *P. falciparum* antigens are present on the surface of infected erythrocytes and bind to the receptors expressed on the VE^[72]^. After invasion, *Plasmodium* modulates endothelial function either by direct adhesion to the EC receptors or by releasing parasite products that can induce EC activation, leading to the disruption of the EC barrier^[73]^. It has also been shown that histones released from merozoites (HeH) stimulate the production of inflammatory mediators by primary human dermal microvascular endothelial cells, supporting the pathogenic role of both host- and pathogen-derived histones in *P. falciparum* caused malaria^[128]^.

Malaria caused by *P. falciparum* is associated with the cytoadherence to endothelial cells through the parasite ligand *P. falciparum* erythrocyte membrane protein 1 (PfEMP1). *P. falciparum*-infected RBCs sequester in blood capillaries through several endothelial cell cytoadherence receptor molecules such as CXCL1, ICAM1, CD36, and VCAM1^[129]^ and release exosome-like vesicles to directly communicate between the parasites^[104]^. These extracellular vesicles and the other abnormal accumulation of metabolites play a critical role in the damage of the blood-brain barrier (BBB) in determining the severity of cerebral malaria (CM) caused by *P. falciparum*. CM is accompanied by coma, seizures, and focal neurological deficits, which contribute to a mortality rate of 15%-20% despite therapy^[130]^. After establishing infection, *Plasmodium* export many proteins, including epoxide hydrolases into the erythrocyte, which results in the alteration of fatty acid composition, leading to perturbed vascular function and sequestration of the parasite in the VE^[74]^. Exportation of PfEMP1 mediates the adhesion of infected erythrocytes to VE and placental syncytioblasts ^[131]^. In addition, a recent study suggests that brain ECs produce low molecular weight growth factors, which stimulate the growth of *P. falciparum in vitro*. These growth factors potentially enhance parasite proliferation in erythrocytes in the brain microvasculature^[75]^.

### Role of exosomes in malaria

Production of exosomes from infected-host cells and *Plasmodium* species during infection correlates with higher malarial disease severity^[39,132]^. Supporting this idea, a study of *P. falciparum* revealed that exosome-like particles released from infected RBCs facilitate cell-to-cell communication among parasites through gene delivery. The *P. falciparum* protein PfPTP2 has been identified as a critical player in this mechanism. This cellular communication pathway promotes the multiplication of sexual forms (gametocytes), which is a key process to maintain malaria infection and increase transmission probability^[104]^. Furthermore, it has been hypothesized that blocking the synthesis of these exosome-like vesicles as a therapeutic target may lead to decreased parasite transmissibility^[104]^.

Exosome production may serve as a means of host protection during malaria infection. In a study of *P. yoelii*-infected mice, parasite protein-containing exosomes were released from reticulocytes, which could induce antigen presentation and elicit a long-term antibody protective immune response when administered as a vaccine in naive mice. The production of IgG antibodies and recognition of the parasite-infected RBCs in response to the vaccination with released exosomes reduces the parasite load and leads to increased survival as well as reticulocytosis^[102]^. Vaccination of mice *in vivo*, as well as *in vitro* in human spleen cells, with CpG adjuvanted *P. yoelii*-infected reticulocyte-derived exosomes (rexPy) induces a spleen-dependent memory response against the parasite infection. This memory response is associated with the activation of spleen cells through rexPy uptake, leading to changes in the distribution of T cell subsets and, more specifically, an increase in memory CD4+ and CD8+ T cells^[103]^. Due to the immunoregulatory action, *Plasmodium* exosome particles are viable candidates in the development of future malaria vaccines^[39]^.

## LEISHMANIASIS

Leishmaniases are a group of neglected tropical diseases caused by infection with parasites belonging to the genus *Leishmania*, which are transmitted by the bite of infected sand flies. It is estimated that one billion people are at risk of infection and about 1.7 million new cases of leishmaniasis occur each year in 102 countries (https://www.who.int/leishmaniasis/resources/who_wer9122/en/). Due to the lack of efficient treatment options or a vaccine, leishmaniasis has become the second largest cause of death among parasitic infections after malaria. Leishmaniasis consists of a spectrum of clinical syndromes and is dependent on the species of infecting parasite. There are three main forms of the disease: localized or disseminated skin lesions [cutaneous leishmaniasis (CL) or diffuse cutaneous leishmaniasis caused by *L. major* and *L. tropica*], mucocutaneous disease (mucocutaneous leishmaniasis caused by *L. Mexicana*, *L. braziliensis*, and *L. amazonensis*), and systemic disease [visceral leishmaniasis (VL) caused by *L. donovani* and *L. infantum*]. Infection is initiated when infected female phlebotomine sand flies take a blood meal, leading to inoculation with infective promastigotes. Promastigotes are phagocytized by macrophages and neutrophils, which transform into and multiply as intracellular amastigotes, which can metastasize to distant organs. The lifecycle is complete when the infected macrophages are ingested by uninfected sandflies, which transform and replicate as promastigotes in the insect gut (https://www.cdc.gov/parasites/leishmaniasis/biology.html).

### Role of the VE in leishmaniasis

The VE is critical for the initiation of inflammatory processes and vascular remodeling, including angiogenesis and lymphangiogenesis, which occur in the inflammatory microenvironments of both VL and CL infections^[133,134]^. Intra- and extracellular parasites attached to the wall of dermal blood vessels and the capillary lumen lead to the development of secondary infections and the spread of the disease, especially in endemic areas^[135]^. During CL, it has been shown that endothelial cells release NO, which counteracts the recruitment of granulocytes and limits the spreading of infection^[135]^. In CL infections, the venous endothelium of skin lesions expresses ICAM-1, which helps in the migration of lymphocytes to the site of inflammation^[78]^. CL infection with *L. major* has been shown to increase the expression of VCAM-1, which mediates the adhesion of mononuclear cells to the endothelial cells^[79]^. Both human and animal leishmanial infections lead to increased levels of vascular endothelial growth factor-A (VEGF-A) and its receptor (VEGF-R) in the skin^[80,81]^. Recently, Weinkopff *et al.*^[133]^ showed that CL infection by *L. major* induces VEGF-A in macrophages in an ARNT/HIF dependent manner, leading to the limitation of inflammation and lymphangiogenesis. The expansion of the lymphatic network promotes lesion resolution, and inhibition of this process enhances the lesion development. In the VL model, *L. donovani*-infected mice aberrantly express neurotrophic tyrosine kinase receptor type-2 (Ntrk2) on splenic endothelial cells, which plays a role in pathologic remodeling of the spleen^[82]^.

### Role of exosomes in leishmaniasis

The role of exosomes in *Leishmania* infection is well studied, revealing that they serve as a key mode of delivery of *Leishmania* virulence factors and effector proteins to host cells during infection^[136]^. Both pathogen and host-derived exosomes have been identified in this process. *Leishmania*-derived exosomes can transport virulence factors into the host macrophages and induce secretion of IL-8 instead of TNF-β^[105]^. Proteomic analysis has revealed that one such virulence factor contained in *L. major* exosomes is the metalloprotease glycoprotein GP63, which regulates protein tyrosine phosphatases (PTPs) and transcription factors (TFs), such as NF-κB, in target macrophages^[108]^. PTPs prevent macrophage activation by inhibiting the secretion of pro-inflammatory IFN-γ, IL-12, and NO^[137,138]^, which are important in host control of parasite infection. These act to modulate the immune response, diminishing inflammation in favor of parasite growth and survival. Exosomes released from *L. donovani*-infected macrophages contain GP63, which proteolyzes Dicer1 in hepatocytes to block miRNA-122, production leading to disease progression^[109]^. Exosomes released from *L. donovani* promastigotes can effectively alter the cytokine response of monocytes through the up regulation of IL-10 and inhibition of TNF-α production. Similarly, it has been observed that monocyte-derived DCs that have been exposed to parasite exosomes have inhibited levels of IL-12p70, TNF-α, and IL-10. Exosome-exposed DCs cannot induce naïve T cell differentiation into mature Th1 cells^[106]^. In contrast, Schnitzer *et al.*^[110]^ showed that that vaccination with *L. major* antigens present in DC-derived exosomes can induce immune-protection against the infection.

*Leishmania* is also able to modify the production and content of exosomes in response to environmental stress (heat shock and pH) that mimic infection. Silverman *et al.*^[105]^ showed that vesicle release from parasites can be increased by three-fold in response to heat shock. Interestingly, Atayde *et al.*^[107]^ showed that *Leishmania* release exosomes within the lumen of the sand-fly midgut, which are ejected in the egested inoculum during by the sand-fly bite. These exosomes lead to exacerbated lesions in *L. major* and *L. infantum* models, possibly due to increased production of inflammatory cytokine IL-17α, and overproduction of IL-4 and IL-10, which are both known to suppress the Th1 responses and play a role in disease susceptibility.

## TOXOPLASMOSIS

Toxoplasmosis is caused by the obligate intracellular protozoan parasite, *Toxoplasma gondii*, which infects healthy and immunocompromised individuals worldwide. It is estimated that more than 11% of the population in the US and 60% of the population throughout the world are infected^[139]^ (https://www.cdc.gov/parasites/toxoplasmosis/epi.html). The transmission of toxoplasmosis mainly occurs through ingesting raw meat containing *T. gondii* cysts (foodborne) or water containing oocysts from feline feces (waterborne). The parasite can also be transmitted congenitally when a woman acquires the infection during pregnancy^[139]^, or very rarely through transplantation of organs^[140]^. Although felines act as definitive hosts for *T. gondii*, it can infect almost all nucleated mammalian and avian cells. The life cycle of *T. gondii* mainly involves an asexual phase within nucleated cells and a sexual phase, which occurs in felines. Ingested oocysts released in feline feces serve as the infectious stage for humans. Toxoplasmosis can cause miscarriage, stillborn infants, or severe central nervous system (CNS) disease; in adults, it can lead to multi-organ involvement including encephalitis, retino-uveitis, and pulmonary disease, especially in immunosuppressed hosts cerebral toxoplasmosis.

### Role of the VE in toxoplasmosis

It is believed that *T. gondii* can modulate the gene expression of brain ECs and promote dissemination through the BBB. *T. gondii* transforms into motile extracellular forms (tachyzoites) that use transcellular or paracellular migration to cross the BBB and infect host cells. In this context, the ECs serves as a replicative niche for the entry of *T. gondii* to the CNS^[83]^. The most common clinical manifestation of toxoplasmosis is retinal infection. Recently, Furtado *et al*^[141]^ demonstrated that tachyzoites can cross the retinal endothelium to establish infection and that blocking this entrance leads to reduced diseased burdens. It has also been observed that *T. gondii* infection leads to induction and activation of cerebral blood vessel ECs^[84]^.

### Role of exosomes in toxoplasmosis

*T. gondii* was the first non-viral protozoan pathogen for which exosomes were identified^[38]^. Although *T. gondii* exosomes are continuously released during infection by tachyzoites, the bulk of released molecules are non-exosome associated excretory/secretory antigens constitute during the acute phase of infection^[114]^. An earlier study conducted by Aline *et al.*^[111]^ revealed exosomes are important in generating protective immunity against *T. gondii* infection; *T. gondii*-pulsed DCs can effectively induce a spleen-derived Th1 immune response that is protective against acute and chronic infection It has also been shown that exosomes secreted by SRDCs induce a protective humoral immune response against the infection in syngeneic and allogeneic mice associated with high levels of IgA antibodies^[112]^. Furthermore, vaccination of mice before pregnancy with exosomes secreted by *T. gondii*-pulsed DCs protects pups from congenital infection due to a robust T cell response^[142]^.

Li *et al.*^[113]^ characterized *T. gondii*-derived exosomes as ~50 nm in size and containing HSP70, CD63, and *T. gondii* surface marker P30. These exosomes were shown to modulate macrophage activation through increased production of IL-12, TNF-α, and IFN-γ and a decrease in IL-10. Mice immunized with these exosomes exhibited cellular and humoral immune responses and were protected against acute infection^[39,113]^. Li *et al.*^[143]^ also reported that *T. gondii* exosomes activate JNK signaling to elicit this innate immune response. Macrophages infected with *T. gondii* release exosomes containing PAMPs, which activate inflammatory responses in adjacent macrophages in a TLR- and myeloid differentiation factor 88 (MyD88)-dependent manner^[53]^. Some *T. gondii* exosomes contain miRNA that may interact and modulate the host cells through gene regulation^[114]^. Taken together, *T. gondii*-derived exosomes displayed significant immunogenic properties that make them viable candidates for vaccine production.

## CHAGAS DISEASE

The hemoflagellate *Trypanosoma cruzi* is the etiologic agent of Chagas disease, also termed American trypanosomiasis. Chronic infection by this parasite is characterized by chronic myocarditis, cardiomyopathy, and vasculopathy, as well as mega-organ syndromes^[144–147]^. It is estimated that ~8 million people are currently infected worldwide and that 20%-30% of those individuals develop sequelae of chronic infection. Chagas disease is the leading cause of heart failure in Latin America^[148–151]^. The parasite exists in four morphological forms: epimastigotes, insect metacyclic trypomastigotes, human trypomastigotes, and intracellular amastigotes^[151]^. Insect stage trypomastigotes (also termed metacyclic trypomastigotes) are the infective stage of the parasite, which are present in the feces of hematophagous triatomine insects, which contaminate wounded skin or mucous membranes^[148,152]^. Metacyclic trypomastigotes invade nucleated host cells to establish the infection. Inside the host cells, they transform and replicate as intracellular amastigotes, eventually differentiating into blood-stage trypomastigotes and exit the host cells to disseminate to multiple organs, including the heart and gastrointestinal tract of the mammalian hosts^[148]^.

### Role of the VE in Chagas disease

*T. cruzi* infects various types of cells including cardiac myocytes, GI-tract, smooth muscle cells, and the VE^[95,153]^. The VE plays a key role in the dissemination of *T. cruzi*, as parasites engage ECs during the initial stages of infection^[85,86]^. As the infection progresses, VE release inflammatory molecules by direct physical disruption of ECs, and parasites undergo trans-endothelial migration with the help of the parasite protease, cruzipain^[85,87,88]^. Cruzipain cleaves the human kininogen into bradykinin, an inflammatory mediator of endothelial permeability ^[85,154,155]^ . Studies have shown that *T. cruzi* induces ECs release of vasoactive molecules endothelin-1, pro-inflammatory cytokines (IL-1β, IL-6, and TNF-α), and thromboxane A2, which trigger the production of iNOS and nitrosative stress^[89–92]^. Moreover, increased levels of TNF-α exacerbate endothelial COX-2/TXA2/TP/superoxide signaling^[92]^. The infection of *T. cruzi* also activates the NF-κB that accumulates in the nucleus and activates many genes specific to endothelial pathophysiology^[95]^.

In addition to the vascular damage, *T. cruzi* invades VE through the secretion of neuraminidase that removes sialic acid from the infected ECs^[156,157]^. Collectively, all of these factors compromise the activity of ECs and cause vasculopathy, as demonstrated by vasospasm, focal ischemia, reduction in the blood flow, increased platelet aggregation, and elevated levels of thromboxane A (2) and endothelin-1^[89,158,159]^. Studies have shown that *T. cruzi*-infected ECs secrete endothelin-1 and interleukin-1beta (IL-1β), which activate extracellular signal-regulated kinases 1 and 2 (ERK1/2) and NF-κB, resulting in the expression of cyclin-D1 in uninfected smooth muscle cells^[93–95]^. The activation of these pathways involved in the interaction between *T. cruzi* and the VE are likely to play a major role in the inflammatory responses after vascular injury and endothelial dysfunction and could be potential targets for therapies to control parasite dissemination^[65,85]^.

It is estimated that 10%-30% of patients infected with *T. cruzi* progress to the chronic stage manifested by cardiomyopathy and prothrombotic/inflammatory status^[160]^. Pathophysiological mechanisms such as activation of the endothelium and microvascular alterations occur during the cardiac damage. It is known that thromboxane A2 increases platelet aggregation and that inhibiting the formation of thromboxane A2 by aspirin alters the course of Chagas disease in both acute and chronic phases^[161]^. Benznidazole, a widely used drug for the treatment of Chagas disease, also acts by preventing endothelial damage caused by *T. cruzi*^[162]^. Cholesterol-lowering drugs such as simvastatin have been shown to decrease the endothelial activation and, in combination with benznidazole, improve the pathophysiological condition of chronic Chagas disease patients^[163]^. Recent discoveries have provided insights into how *T. cruzi* escapes the BBB and rapidly migrates across the ECs without disrupting the integrity of the monolayer or altering the permeability. Coates *et al.*^[85]^ identified that this process is facilitated by bradykinin and CCL2, which may be considered in the development of new therapeutic strategies for Chagas disease.

### Role of exosomes in Chagas disease

Parasite and host cell exosomes play a role in the pathogenesis in Chagas disease. The release of an elevated number of exosomes is essential for host-parasite interaction, intercellular communication, and enhanced parasite survival^[120]^. The infection of *T. cruzi* induces blood cells to release exosomes through a Ca^2+^-dependent manner^[164]^. The released exosomes protect extracellular trypomastigotes from complement-mediated lysis by binding to C3 convertase on the *T. cruzi* surface and inhibiting C3 cleavage^[115,117]^. Exosomes also aid the parasites to invade the host cells through the expression of TGF-β. The communication between cells takes place through the release of exosomal contents including cytokines, peptides, hormones, microRNA, and numerous bioactive substances, which act as a function of innate immunity^[165,166]^. Exosomes released by *T. cruzi* promote cell invasion and parasite survival by modulating the innate immune system and producing several virulence factors including the glycoprotein 85 (gp85), trans-sialidase, phosphatase, and the soluble proteins^[116,119,120,167]^. Thus, the exosomes released by the host cells and parasites during infection play a vital role in the invasion of the innate immune system, parasite survival, and the establishment of infection in Chagas disease.

## HUMAN AFRICAN TRYPANOSOMIASIS

Human African trypanosomiasis (HAT) is caused by two subspecies of *Trypanosoma brucei: T.b. gambiense* that leads to the chronic form of HAT known as West African trypanosomiasis and *T.b. rhodesiense* that leads to the acute form of HAT known as East African trypanosomiasis^[168,170]^. A third subspecies of *T. b. brucei* infects cattle and very rarely infects the human host. HAT is transmitted to mammalian hosts by the bite of infected tsetse flies. During a blood meal, the metacyclic trypomastigotes are injected into the skin of the host, eventually entering the lymphatic and blood vessels. As parasites transform into blood trypomastigotes, they are disseminated throughout the body. The life cycle is completed when trypomastigotes infect feeding tsetse flies, wherein they transform and replicated into insect stage parasites^[168,171]^. Unlike the other protozoan parasites, the entire life cycle of African trypanosomes consists of extracellular stages, which alternately infect mammalian and insect hosts (CDC.gov, https://www.cdc.gov/parasites/sleepingsickness/biology.html). Although *T. brucei* infection occurs through the hemolymphatic stage in the initial systemic stage, the second phase is mainly characterized as a central nervous system disease, due to parasite invasion of brain tissue, leading to the altered sensorium, seizures, coma, and death. These symptoms are the reason that HAT is also referred to as African sleeping sickness.

### Role of the VE in HAT

Bloodstream forms of *T. brucei* multiply to high density and eventually invade the central nervous system through the penetration of the VE. Although the mechanism through which the *T. brucei* cross the BBB are yet to be fully understood, it has been shown that *T. brucei* uses a multi-step process using the host derived factors including the cytokines IFNα/β, IFNγ, TNF, ICAM-1, and CXCL10^[97,172]^. During infection, the VE cells are activated by the translocation of NF-κB, due to action of parasite trans-sialidase, to the nucleus and the induction pro-inflammatory cytokines such as TNF-α, IL-6, and IL-8. This process plus the induction of other soluble factors such as the adhesion molecules (ICAM-1, E-selectin, and VCAM-1)^[100]^ culminates in leukocyte recruitment and transmigration of trypanosomes from the VE to the CNS^[96,98]^. Studies have shown that *T. brucei* infection enhances the eNOS protein expression, and enhanced NO production leads to elevated vasodilation and vascular permeability facilitating parasite invasion into the surrounding tissues and the central nervous system^[173]^.

Parasite phospholipase C, protein kinase, and the parasite cysteine protease brucipain also participate in transmigration of trypanosomes into the CNS^[101,174]^. Furthermore, it has been shown that *T. brucei* crosses the VE of cerebral blood vessels of mice through interaction with laminin 8 of the ECs^[97]^. The transmigration of *T. brucei* through the vascular endothelium also depends on the calcium and the papain-like cysteine proteases^[101]^. Collectively, this shows that interaction with the VE depends on various factors that are essential to penetrate the BBB and infect the CNS.

### Role of exosomes in HAT

The progression of HAT is modulated by several factors including macrophage hyper-activation, uncontrolled production of TNF, and the transfer of virulence factors by exosomes^[123,175]^. Host-derived exosomes play a major role in host defense and are targeted as vaccine candidates, whereas parasite-derived exosomes transduce signal(s) to the host cells to establish infection^[39,176,177]^. A study has shown that a spliced ladder RNA (SL RNA) is present in the exosomes of *T. brucei* that is essential in these parasites for the formation of all mature mRNA. The cells secreting these SL RNA-containing exosomes affect the social motility of these parasites ^[178]^. The bloodstream form of the parasite is responsible for anemia and tissue damage in the mammalian host^[124,179]^. This immunopathological outcome is due to several proteins released from the exosomes that lead to sequential activation of the innate and acquired immune responses^[180]^. The parasites secrete several molecules through exosomes to gain access to the host cells. Likewise, *T. brucei* exosomes contain 156 proteins from diverse functional classes ^[123]^. One study shows that *T. brucei* exosomes fuse with mammalian erythrocytes and causes rapid clearance of erythrocytes and promotes anemia^[123,124]^. Thus, the exosomal components (proteases) of the trypanosome could be the promising targets to control sleeping sickness^[181]^.

## CONCLUDING REMARKS

Here, we discuss the important roles played by VE and exosomes in some major protozoan parasitic diseases. Exosomes serve as a carrier of effector molecules that modulate the host immune response in establishing infection. The content of exosomes provides an effectual means to control the protein expression in both parasite and host cells. While parasite-derived exosomes play a key role in establishing infections through intercellular communication and signaling mechanisms, the host-derived exosomes also play a major role in the host-defense mechanism. Understanding the mechanism of the exosomal component on the host immune system in causing parasitic disease may help in the development of a novel approach of diagnostic tools and treatment. Further research on exosomes is necessary to search for the candidate vaccine and drug development.

The VE provides effective immunological homeostasis that controls the inflammatory response, mainly through the production of cytokines. The VE is an important target of parasite invasion and the parasite interaction on the VE is responsible for the development of clinical manifestations. The trans-endothelial migration of parasites is the major key step in establishing infection. Thus, more experimental studies are needed to provide insights on the interaction of blood parasites and VE, trans-endothelial migration, and the role of endothelial cytokine mediators in parasite dissemination. A better understanding may reveal the way to find more anti-parasitic regimens.

**Figure 1.**
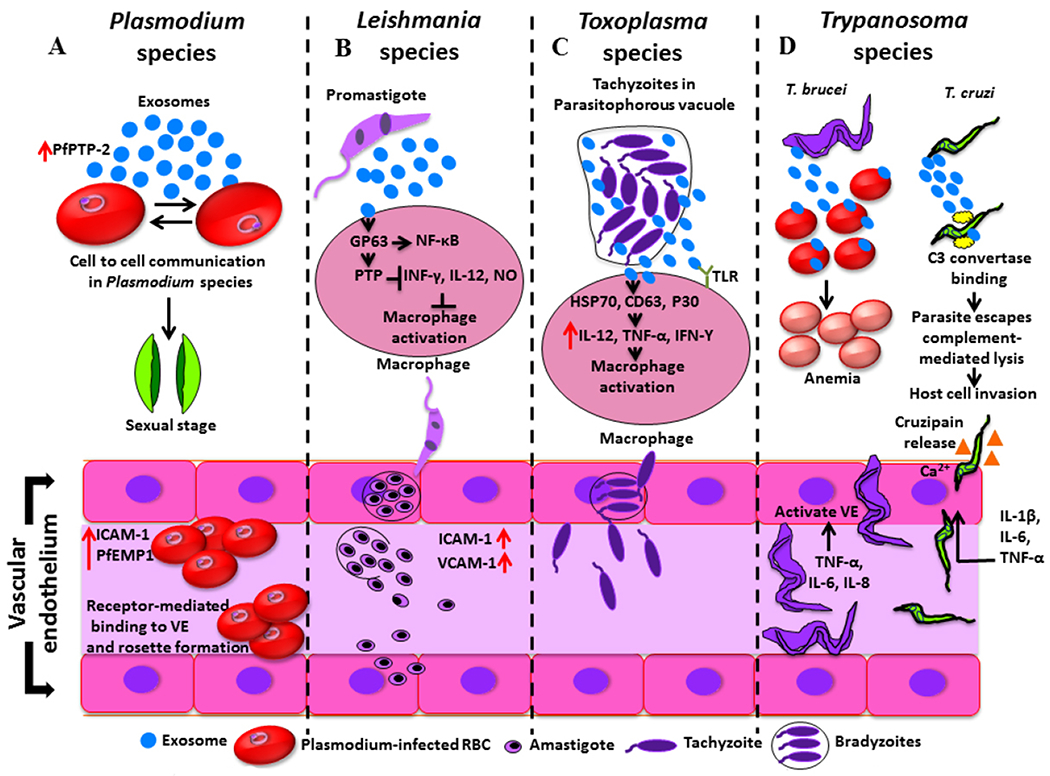
Schematic representation of cellular and molecular mechanisms played by vascular endothelium (VE) and exosomes in *Plasmodium, Leishmania, Toxoplasma,* and *Trypanosoma* spp. Infection. A: *P. falciparum* protein PfPTP-2 released through the exosomes from infected red blood cells (RBCs) facilitates cell-to-cell communication and promotes the differentiation of sexual forms of the parasites. *P. falciparum* erythrocyte membrane protein-1 (PfEMP1) and intercellular adhesion molecule-1 (ICAM-1) mediate the adhesion of infected erythrocytes to the VE and placental syncytioblasts; B: *Leishmania* parasites transport glycoproteins such as GP63 into the host cells through exosomes and regulate the protein tyrosine phosphatases (PTPs) and transcription factors such as NF-κB in macrophages. The PTPs prevent macrophage activation by inhibiting the secretion of IFN-γ, IL-12, and nitric oxide (NO). *Leishmania* infection also increases the expression of intercellular adhesion molecule-1 (ICAM-1) and vascular cell adhesion molecule-1 (VCAM-1) to initiate an inflammatory response after migrating the mononuclear cells and lymphocytes to the endothelial cells; C: *T. gondii* parasites release exosomes, which contain HSP70 and CD63, as well as the *T. gondii* surface marker P30. These exosomes induce the production of IL-12, TNF-α, and IFN-γ and modulate macrophage activation; D: *T. brucei* releases exosomes that are deposited and fused to RBCs. The virulence factors of exosomes result in RBC membrane alteration and anemia. In addition, *T. brucei* activates the vascular endothelial cells by producing TNF-α, IL-6, and IL-8. The exosomes of *T. cruzi* contain C3 convertase binding protein, which helps the parasites to escape the complement-mediated lysis. *T. cruzi* releases cruzipain and invades vascular endothelium through a Ca^++^-dependent mechanism

To summarize, both the VE and exosomes regulate the entry of parasites, their multiplication, signaling between the parasite and host cells, and dissemination to the other organs of the hosts. In addition, the VE and exosomes modulate both the innate and adaptive immune responses and maintain the integrity of the inflammatory process (summarized in Figure 1 and Tables 1 and 2). However, further studies are needed for a thorough understanding of the mechanisms and roles played by the VE and exosomes in parasite survival and disease progression. The novel mechanisms regulated by the VE and exosomes can be considered as potential therapeutic targets to treat and control these human protozoan diseases.

## Figures and Tables

**Table 1.** Roles and mechanisms of vascular endothelium in malaria, leishmaniasis, toxoplasmosis, Chagas disease, and HAT

Parasite name	Disease	Role of VE	Ref.
*Plasmodium* spp.	Malaria	Expresses receptors for *Plasmodium* antigens	[72,73]
		Reservoir for epoxide contains lipid signaling molecules and helps in multiplication of parasites	[74]
		Produces low molecular weight growth factors, which enhance the parasite proliferation	[75]
*Leishmania* spp.	Leishmaniasis	Reservoirs for intra- and extracellular parasites	[76]
		Releases nitric oxide (NO) and limits the spread of the disease	[77]
		Expresses ICAM-1 in skin lesions in cutaneous disease, which helps lymphocyte migration s to sites of inflammation	[78]
		Increases expression of VCAM-1, VEGF-A and VEGF-R in the skin lesions in cutaneous disease	[79–81]
		Splenic endothelial cells express Ntrk2, helps in the pathological remodeling of the spleen in visceral disease	[82]
*Toxoplasma*.	Toxoplasmosis	Serves as replicative niche and provides the entrance to CNS	[83]
		*T. gondii* infection leads to activation of cerebral endothelial cells, facilitating the spreading of the disease	[84]
*Trypanosoma* spp.	Chagas disease	Key role in the dissemination of parasites to the other organs	[85,86]
		Produces various inflammatory molecules leading to trans-endothelial migration	[85,87,88]
		Releases vasoactive molecules such as endothelin-1 and pro-inflammatory cytokines IL-1β, iL-6, TNF-α, and thromboxane A2 leading to the production of iNOS	[89–92]
		Produces endothelin-1 and IL-1β, activated ERK1/2 and NF-κB, resulting in the induction of Cyclin-D1 in uninfected cells	[93–95]
*Trypanosoma* spp.	Human African trypanosomiasis	Serves as replicative niche	[96,97]
		Produces inflammatory cytokines such as TNF-α, IL-6, and IL-8	[98,99]
		Induces the production of ICAM-1, E-selectin, and VCAM-1 to facilitate parasite migration into the central nervous system (CNS)	[96,98,100]
		Facilitates parasite transit across the endothelium of cerebral blood vessels by the production of laminin-8, calcium, and papain-like cysteine proteases	[97,101]

VE: vascular endothelium; HAT: Human African trypanosomiasis; ICAM-1: intercellular adhesion molecule-1; VCAM-1: vascular cell adhesion molecule-1; VEGF-A: vascular endothelial growth factor-A; VEGF-R: vascular endothelial growth factor receptor; IL: interleukin

**Table 2.** Roles and mechanisms played by exosomes in malaria, leishmaniasis, toxoplasmosis, Chagas disease, and HAT

Disease	Exosomal Factors	Cell origin	Mode of Action	Ref.
Malaria	Parasitic components (protein, lipid, RNA, DNA)	Infected reticulocytes	Induces antigen presentation and elicit a long-term antibody protective immune response, increase memory CD4+ and CD8+ T cells	[102,103]
	Pathogen genes	Infected RBCs	Facilitates cell-to-cell communication between parasites, promote differentiation to sexual forms	[104]
Leishmaniasis	Virulence factors and effector proteins	Parasite	Induces secretion of IL-8 over TNF-α in host macrophages	[105]
			Alters the cytokine response of monocytes through upregulating IL-10 and inhibiting TNF-α production	[106]
			Inhibits IL-12p 70, TNF-α, and IL-10 cytokine functions in monocyte-derived DCs and prevent DC-induced naïve T cell differentiation into mature Th1 cells	[107]
	GP63	Parasite	Exacerbates lesions due to increased production of inflammatory cytokine IL-17α and over the induction of IL-4 and IL-10	[108]
		Infected macrophages	Regulates PTPs and TFs in target macrophages	[109]
	Antigenic proteins	Infected DCs	Cleaves Dicer1 in hepatocytes to block miRNA-122 production, causing a decreased serum cholesterol level	[110]
Toxoplasmosis	Antigenic proteins	Infected DCs	Induces protective spleen-derived Th1 and humoral immune responses with high levels of IgA antibody	[111,112]
	Exosome	Parasite	Modulates macrophage activation through increased production of IL-12, TNF-α, and IFN-γ and a decrease in IL-10	[113]
	PAMPs	Parasite	Induces protective cellular and humoral immune responses	[53]
	miRNA	Parasite	Activates inflammatory responses in nearby macrophages in a TLR- and MyD88-dependent manner. Interact and modulate host cells through gene regulation	[114]
Chagas disease	Exosome	Infected blood cells	Protects extracellular parasites from complement-mediated lysis by binding the C3 convertase on the parasite surface and inhibiting C3 cleavage	[115–117]
	Virulence factors and soluble proteins	Parasite	Helps parasites to invade host cells through the expression of transforming growth factor-beta (TGF-β)	[118]
	Complement regulatory and inhibitory proteins	Parasite	Enhances cell invasion and parasite survival by invading the innate immune system	[119,120]
			Avoids the complement system and increase the invasion of host cells	[121,122]
HAT	Virulence factors and proteins (SRA)	Parasite	Activates the innate and acquired immune responses and induces rapid clearance of erythrocytes to cause anemia and tissue damage	[123,124]

HAT: Human African trypanosomiasis; IL: interleukin; PAMPs: pathogen associated molecular patterns; TLRs: toll-like receptors; PTPs: protein tyrosine phosphatases; TFs: transcription factors
